# Dihydromyricetin induced lncRNA MALAT1*-*TFEB-dependent autophagic cell death in cutaneous squamous cell carcinoma

**DOI:** 10.7150/jca.32807

**Published:** 2019-07-10

**Authors:** Miduo Tan, Bin Jiang, Haihua Wang, Wei Ouyang, Xiang Chen, Taoli Wang, Dan Dong, Shun Yi, Jiansheng Yi, Yan Huang, Manling Tang, Yan Xiao, Zuiming Jiang, Wei Zhou

**Affiliations:** 1 Surgery Department of Galactophore, The Affiliated Zhuzhou Hospital of Xiangya Medical College CSU, Zhuzhou 412000, China.; 2 Department of Plastic Surgery, The Second Hospital University of South China, Hengyang, 421001, China.; 3 Department of Burns and Plastic Surgery, The Affiliated Zhuzhou Hospital of Xiangya Medical College CSU, Zhuzhou 412000, China.; 4 Department of Oncology, The Affiliated Zhuzhou Hospital of Xiangya Medical College CSU, Zhuzhou 412000, China.; 5 Clinical Laboratory Center, The Affiliated Zhuzhou Hospital of Xiangya Medical College CSU, Zhuzhou 412000, China.; 6 Department of Pathology, The Affiliated Zhuzhou Hospital of Xiangya Medical College CSU, Zhuzhou 412000, China.; 7 Department of Dermatology, The Second Hospital University of South China, Hengyang, 421001, China; 8 Department of General surgery, The Affiliated Zhuzhou Hospital of Xiangya Medical College CSU, Zhuzhou 412000, China; 9 Department of Otolaryngology, The Affiliated Zhuzhou Hospital of Xiangya Medical College CSU, Zhuzhou 412000, China

**Keywords:** Dihydromyricetin, autophagy, TFEB, MALAT1, Cutaneous squamous cell carcinoma

## Abstract

Cutaneous squamous cell carcinoma (CSCC) is the second most common skin cancer. Dihydromyricetin (DHM), a Rattan tea extract, has been shown to have antitumor activity with no obvious toxicity to normal cells in vitro and in vivo. However, its efficacy in the treatment of CSCC and the underlying antitumor mechanism has not been fully elucidated yet. In our study, DHM increased autophagic flux in the A431 cells, as evidenced by the upregulation of LC3-II and downregulation of P62/SQSTM1. Moreover, the pharmacological or genetic blocking autophagy decreased DHM-induced cell death, indicating DHM triggered autophagic cell death in A431 cells. Specifically, DHM induced TFEB^(Ser142)^ de-phosphorylation, activated TFEB nuclear translocation and increased of TFEB reporter activity, which contributed to the expression of autophagy-related genes and subsequent initiated autophagic cell death in A431 cells. Importantly, DHM decreased lncRNA MALAT1 expression and MALAT1 overexpression abrogated the effects of DHM on TFEB-dependent autophagy both in vitro and in vivo. Taken together, DHM induces CSCC cell death via inducing excessive autophagy, which is mediated through the *MALAT1*-TFEB pathway. Therefore, DHM may be beneficial for the development of chemotherapy for CSCC.

## Introduction

Cutaneous squamous cell carcinoma (CSCC), is widely considered as one of the most common skin cancers, secondary only to basal-cell carcinoma and represents between 20%-50% of all known skin cancers worldwide 1. The clinical treatment with the surgery shows efficiency only in early-stage and well-defined CSCC, while elder patients with comorbid conditions and those on immunosuppressants or anticoagulants may be ineligible for surgery 2. Moreover, eyelid, lip or ear lesions need maximum tissue preservation and thus, surgery becomes inappropriate or may lead to poor cosmetic outcome. Treatment with cryotherapy, miquimod and 5-fluorouracil (5-FU) are associated with poor outcome and high recurrence rate 3. Thus, treatment of CSCC still represents one of the most challenging problems in clinic, and searching novel anticancer agents with high efficient and low side effects is urgently needed in drug development.

Autophagy involves the degradation of long-standing cytosolic proteins and organelles as well as material recycling for maintaining cellular component quality. However, exaggerated autophagy leads to programmed cell death of type II; this is because mitochondria and other survival molecules are intensely degraded 4. Autophagy in cancer also has a dual role, tumor promotion and suppression 5. Certain cancer type triggers autophagy to provide nutrients in order to overcome the chemotherapeutic stress. However, under certain circumstances activation of autophagy upon anticancer drug treatment can also trigger a lethal type of autophagy termed autophagic cell death 6. Understanding the interplay between cell death and autophagy in tumors is crucial to identify new targets for CSCC therapy. Thus, the ability of anti-cancer drugs to increase or decrease autophagic flux in human CSCC cells should be determined.

Transcription factor EB (TFEB), a master regulator for lysosomal biogenesis, modulates lysosomal biogenesis and autophagy by positively regulating genes belonging to the Coordinated Lysosomal Expression and Regulation (CLEAR) network 7,8. TFEB is a member of the basic helix-loop-helix leucine-zipper family that recognizes a 10-base pair motif (5′-GTCACGTGAC-3′) enriched in the promoter regions of numerous autophagy-lysosomal genes 9. Accumulated evidence has led to the integrating hypothesis that activation of TFEB transactivates genes necessary for autophagosome formation, autophagosome-lysosome fusion, and cargo degradation 10. Importantly, under stress full conditions such as chemotherapy treatment, an intricate interplay between the homeostatic TFEB and autophagy pathways may occur in cancer cells that will ultimately dictate their fate between cell death or survival.

Dihydromyricetin (DHM), a natural flavonoid, is an active component of extracts from Ampelopsis grossedentata, exerts anti-inflammatory, hypoglycemic, anti-oxidant, anorexiant, anti-bacterial, anti-photo-aging, anti-allergic, and anti-acne effects^11^.Remarkably, this compound has recently attracted considerable attention because of accumulating data demonstrating its strong inhibitory effect on colorectal carcinoma^12^, hepatocellular carcinoma^13^, and lung cancer^14^. It has been documented that DHM induces apoptosis in human melanoma cells by increasing cleaved PARP and the ratio of Bax to Bcl-2 protein *in vitro* 15. The objectives of the present study were to systematically evaluate DHM as a potential chemopreventive and therapeutic candidate against CSCC progression, and to elucidate the underlying cellular and molecular mechanisms of DHM actions. Here, our results demonstrated that DHM exposure led to induction of autophagic cell death through modulation of the MALAT1/TFEB pathway in A431 cells, and provided experimental evidence to support the future development of DHM as an effective and safe candidate agent for the prevention and/or therapy of CSCC.

## Materials and Methods

### Materials

DHM, Chloroquine (CQ), and 3-methyladenine (3-MA) were purchased from Sigma-Aldrich (St. Louis, MO). CQ was dissolved in distilled water, while the other reagents were dissolved in dimethyl sulfoxide (DMSO), and further dilutions were made with distilled water. DMSO kept at concentrations less than 0.1% had no obvious effect on the cells.

### Cell culture and DHM treatment

A431 cells were obtained from the American Type Culture Collection (Manassas, VA). Cells were cultured in DMEM with 10% heat-inactivated fetal bovine serum, and maintained in a 37°C humidified atmosphere containing 5% CO_2._ The cells were passaged by dissociation in 0.25% trypsin-EDTA solution (Gibco) for 3 min after they reached 80-90% confluency. DHM was dissolved in DMSO to obtain a 100 mM stock solution and was added directly to the medium at different concentrations of 25, 50 or 100 μM for 24 h.

### Animal experiments

Balb/c nude mice (4 weeks of age) were purchased from Vital River Laboratory Animal Technology Corporation (Beijing, China). They were housed individually in stainless steel cages at a constant temperature (25°C) and a 12-h day/night cycle. The day before the experiment the animals were fasted overnight and were allowed free access to water. To investigate the role of the MALAT1-TFEB pathway in melatonin-induced autophagy in *vivo*, MALAT1 overexpression (OP) and wild-type (*WT*) A431 cells (2×10^6^ in 200 μL PBS) were injected subcutaneously into the left flank of each mouse. After 1 week of tumor induction, the animals were divided randomly into another four groups with 10 mice in each group: *WT*+ normal saline, *WT*+DHM (150 mg/kg, i.p.), MALAT1-OP + normal saline, and MALAT1-OP +DHM(150 mg/kg, i.p.) every day for 40 consecutive days. The tumor volume was measured every 5 days for 40 days. Tumor volume was calculated according to the formula 0.5 × length × width^2^. At the end of the experiment, the tumor tissues were collected for further analysis. The studies were carried out in strict accordance with the recommendations in the Guide for the Care and Use of Laboratory Animals of the National Institutes of Health. The protocol was approved by the Ethics Committee of Animal Experiments of Central South University.

### Cell death assay

A431 cells were plated in the 6-well plates (5×10^5^ cells per well) and incubated for 24 h. After being treated with DHM, the cells were detached with 300 μl of a trypsin-EDTA solution. The suspension of the detached cells was centrifuged at 300 g for 5 min. Then, the pellet was combined with 800 μl trypan blue solution and dispersed. After staining for 3 min, the cells were counted using an automated cell counter. The dead cells were stained blue. The cell mortality (%) is expressed as percentage of the dead cell number/the total cell number.

### Lactate dehydrogenase (LDH) assay

Cell death was also evaluated via the quantification of plasma membrane damage that resulted in the release of lactate dehydrogenase (LDH). The amount of LDH released into the cell culture supernatant was detected by an LDH cytotoxicity assay detection kit (Beyotime, China) following the manufacturer's instructions.

### Immunocytochemistry

Cells grown on cover slips were treated as indicated, washed with PBS twice, and then fixed with 4% PFA for 30 min. After a further three TBST washes, the fixed cells were permeabilized with 0.25% Triton X-100 in PBS for 10 min at room temperature. The cells were blocked in blocking buffer (1% BSA, 5% heat-inactivated donkey serum in PBS) for 1 h at room temperature, and subsequently incubated with primary antibodies overnight at 4°C. The fixed cells were incubated with anti-TFEB (1:500, Bethyl Laboratories, A303-673A) in immuno staining dilution buffer at 4°C overnight. The cells were then rinsed and incubated with appropriate Alexa Fluor 568-conjugated secondary antibodies for 1 h, followed by an additional three 10 min TBST washes. The cells were then counterstained with DAPI Staining Solution and mounted on glass slides using Anti fade Mounting Medium. The stained samples were examined using a Zeiss confocal laser scanning microscope equipped with 63× or 40× oil objective.

### Nuclear and cytoplasmic protein extraction

Nuclear-cytoplasmic fractionation was conducted using NE-PER™ Nuclear and Cytoplasmic Extraction Reagents according to the manufacturer's instructions. To assess the purity of the fractionation, the cytoplasmic and nuclear fractions were confirmed by immunoblotting using anti-ACTB as a cytoplasmic marker and anti-histone H3 as a nuclear marker, respectively.

### Western blot analyses

Tumors were harvested after all mice were sacrificed. The tissue samples were homogenized and sonicated in RIPA buffer on ice. Tissue lysates were then centrifuged at 12,000 g for 15 min at 4 °C to collect the supernatant. Total cell lysates were extracted with RIPA buffer supplemented with protease inhibitor cocktail following the indicated treatments. Subsequently, the lysates were incubated for 30 min on ice and centrifuged at 12,000× g for 10 min at 4°C to isolate the supernatants containing the total protein. Then, the protein concentration was determined by BCA assay. Fifty micrograms of protein per lane was separated by 8-12% SDS-PAGE and then transferred to PVDF membranes. After being blocked in 10% non-fat dry milk for 1 h at room temperature, the blots were developed with a series of antibodies overnight at 4°C. The primary antibodies used are listed in Table S1. The blots were then rinsed and hybridized with corresponding HRP-conjugated anti-mouse or anti-rabbit secondary antibodies for 1 h at room temperature. Finally, the membranes were then washed and the visualized using a Luminata Forte Western HRP Substrate and the bands were quantified with Image J software.

### RT-PCR

Total RNA was extracted using TRIzol reagent, and cDNA synthesis was performed using the AffinityScript QPCR cDNA Synthesis Kit with 1 mg of total RNA and random primers. The resulting RT product was expanded using the NovoStart® SYBR qPCR Supermix and data collection were performed with an iQ5 Real-Time PCR Detection System system. The primers used for the amplification of the indicated genes are listed in Table S2.

### Luciferase Reporter Assays

Transient transfection of A431 cells was performed using the TransFectin reagent (Bio-Rad, Hercules, CA, USA). Constructs used were the FHRE-Luciferase reporter and the TFEB expression construct. Inducible activation of TFEB was performed through transfection of the HA-TFEB-WT-ER plasmid. The HA- TFEB-WT-ER fusion protein is constitutively expressed but is inhibited unless exposed to a modified ligand for the oestrogen receptor (ER), 4-hydroxy-tamoxifen (4-OHT). A431 cells were transfected using the TransFectin reagent (Bio-Rad, Munich, Germany) with 1 *μ*g of HA-TFEB-WT-ER plasmid. Activation of the accumulated TFEB protein was induced by treatment with the ER ligand 4-OHT 1 h before DHM treatment. The luciferase reporter activity was measured using a commercially available luciferase assay system (Promega). Transfection efficiency was normalised by *β*-galactosidase activity.

### RNA immunoprecipitation (RIP) assay

RIP assay was performed using the Magna RIP RNA-Binding Protein Immunoprecipitation Kit (Millipore, Billerica, MA, USA). Cells were collected and lysed using an RIP lysis buffer containing a protease inhibitor cocktail and an RNase inhibitor. Next, 100 μl of the whole-cell extract was incubated with RIP buffer containing A + G magnetic beads conjugated with human TFEB antibody or negative control normal mouse IgG (Millipore) for 2 h at 4 °C. Next, 10% of the RIP product and 10% of the cell lysate input was used for western blot analysis while the remaining RIP products were subjected to RIP qRT-PCR for RNA enrichment determination.

### Transfection

siRNAs for TFEB were purchased from Santa Cruz Biotechnology along with control siRNA and siRNA Transfection Reagent. A431 cells were transfected with 100 pM siRNA according to the manufacturer's protocol. At 24 h post-transfection, the cells were exposed to 100 μM DHM for 24 h.

### Lentiviral vector infection

Human MALAT1 full‐length complementary DNA (cDNA) was amplified using PCR. The MALAT1 sequences were designed and the objective products were subcloned into pcDNA 3.1 GenePharma (Shanghai, China). The constructed vectors and the lentivirus packaging vectors (pMD2.G) were recotransfected into the cells 16.

### Statistical analysis

Each experiment was performed independently at least three times. The data are expressed as the mean ± SEM. One-way ANOVAs were used for comparing at least 3 groups. In all analyses, a P value less than 0.05 was considered statistically significant.

## Results

### DHM triggers autophagic cell death in A431 cells

**Figure 1:**
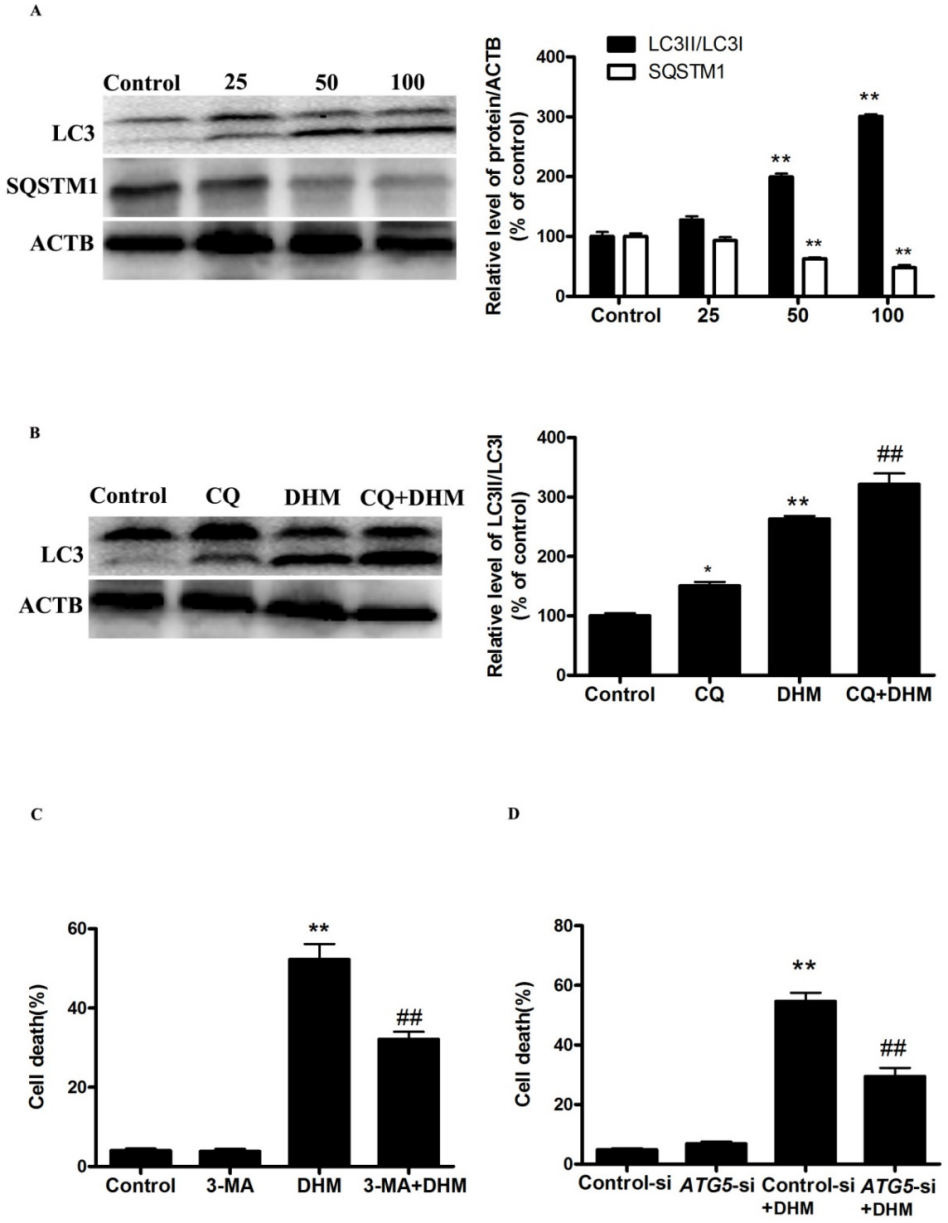
DHM induces autophagic cell death in A431 cells. (A) A431 cells were treated with different concentrations of DHM for 24 h, and the expression levels of the autophagy-associated proteins LC3-I/II, and p62/SQSTM1 were then assessed by western blotting. A431 cells were pretreated with 10 µM CQ for 2 h and incubated with 100 µM DHM for another 24 h. (B) The LC3II levels were then assessed by western blotting; A431 cells were preincubated with (C-D) 3-MA (2 mM) for 2 h or *ATG5*-si for 24 h and then treated with 100 µM DHM for another 24 h, then Trypan blue assay used to assess cell death. **P<0.01 versus the control group, ^##^P< 0.01 versus the DHM (100 µM) group (n=6).

To investigate whether autophagy is involved in the cytotoxicity of DHM, we first examined the processing of full-length LC3-I to LC3-II, a hallmark of autophagy, in DHM-treated A431 cells. DHM increased the protein levels of LC3-II in a dose-dependent manner (Fig. 1A). We also observed an evident dose-dependent decrease in the p62/SQSTM1 protein levels in A431 cells that were treated with DHM, confirming that autophagic flux was intact in the DHM-treated cell (Fig. 1A). To further confirm autophagy flux inducing effect of DHM, we incubated A431 cells with CQ, which increases lysosomal PH and thereby inhibits lysosomal degradation, for 2 h prior to exposure to 100 µM DHM. We found that a CQ challenge resulted in increased LC3II expression in the cells treated with 100 µM DHM, and these findings demonstrated that DHM treatment induced autophagic flux in A431 cells (Fig. 1B). Because the manipulation of autophagy may be involved in the efficacy of anticancer therapeutics, we were eager to assess the effect of DHM-elicited autophagy on the survival of A431 cells. As shown in Figure S1A, 3-MA, an autophagy inhibitor, reduced the percentage of autophagy from 320% to 80%, and the percentage of cell death from 52% to 32% (Fig.1C and S2A). In agreement with the data derived from pharmacological inhibitor, knockdown of ATG5 by siRNA also efficiently protected against DHM-induced cell death (Fig. 1D, S1B and S2B). These results show that DHM induces autophagic cell death in A431 cells.

### TFEB signaling was implicated in autophagy induction by DHM in A431 cells

**Figure 2:**
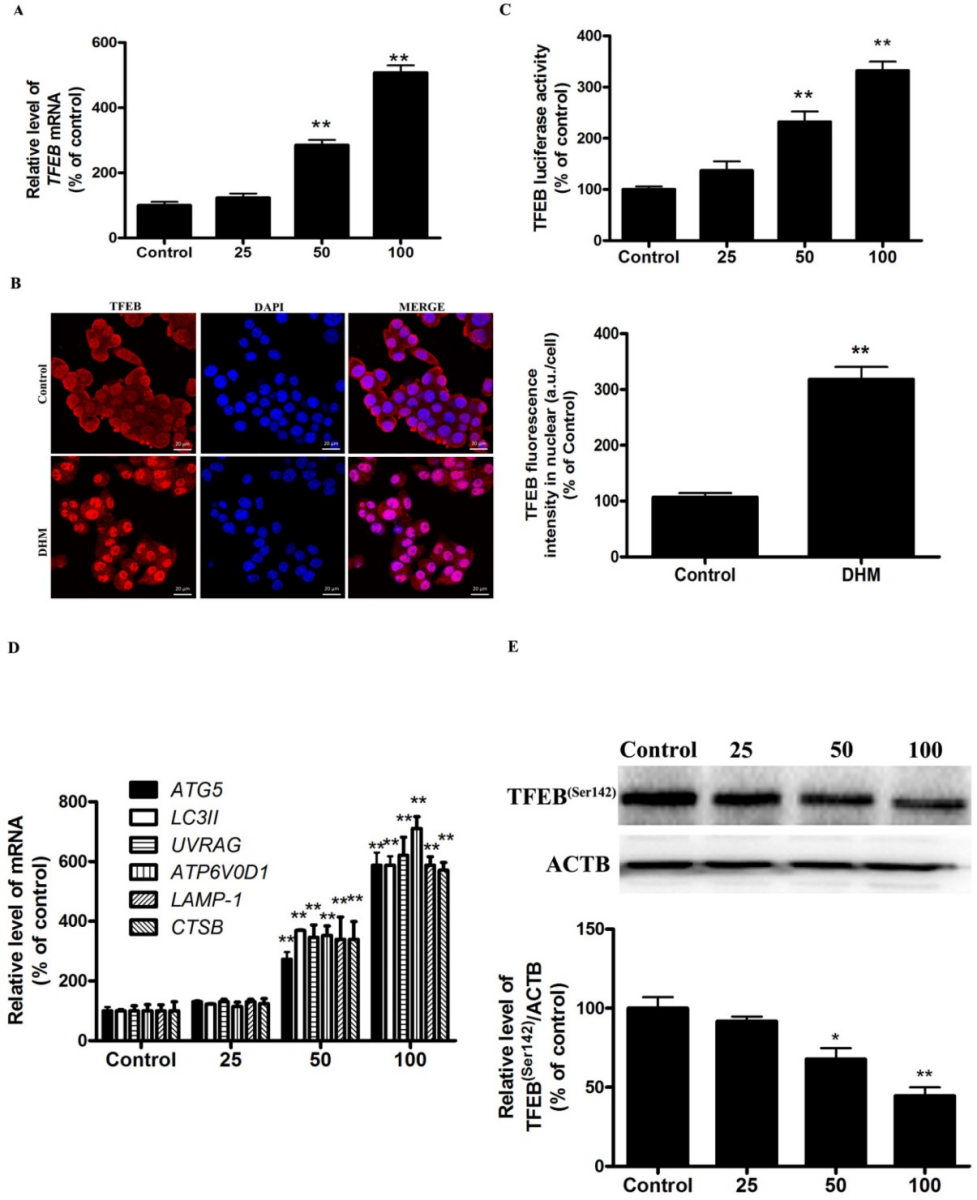
DHM increases TFEB activity in A431 cells. (A) A431 cells were treated with different concentrations of DHM for 24 h, and the mRNA level of TFEB were then analyzed by RT-PCR; (B) Immunofluorescence of A431 cells incubated with anti-TFEB antibody and DAPI after DHM (100 µM) treatment for 24 h; (C) A431 cells were transfected with TFEB-luciferase expression vector and incubated with different concentrations of DHM. The luciferase activity was then measured. (D) The mRNA levels of TFEB-target genes were measured using RT-PCR. (E) phosphorylation of TFEB protein was detected by western blotting; *P<0.05, **P<0.01 versus the control group (n=6).

More recently, TFEB, a master regulator of lysosome biogenesis and autophagy, has emerged as leading factors in human disease pathology. Based on these results, we investigated whether the TFEB are involved in the action of DHM in A431 cells. Here, TFEB mRNA expression increased significantly after exposure to different concentrations of DHM for 24 h (Fig. 2A). Nuclear (ie, transcription-dependent) and cytoplasmic (ie, transcription-independent) functions were described for TFEB in the promotion of autophagy; we next sought to determine the subcellular localization of TFEB upon DHM treatment using immunofluorescence. Interestingly, the data obtained by immunofluorescence revealed that TFEB translocated into the nucleus following DHM exposure (Fig. 2B). Moreover, the entry of TFEB into the nucleus was accompanied by the upregulation of “TFEB-target genes”. As expected, DHM significantly increased the TFEB luciferase activity and the mRNA abundance of 6 tested genes, including ATG5, MAP1LC3B, UVRAG, ATP6V0D1, LAMP1, and CTSB (Fig. 2C-D). Taken together, these results indicated an important role for TFEB signaling in DHM-induced autophagy in A431 cells. It is reported that the function and activity of TFEB is mainly regulated by phosphorylation-dependent nuclear-cytoplasmic shuttling. We then measured whether a post-translational modification of TFEB was required for triggering autophagy. Figure 2E shows that DHM significantly decreased Ser142 phosphorylation of TFEB protein.

### TFEB gene silencing inhibits DHM-induced autophagic cell death in A431cells

**Figure 3:**
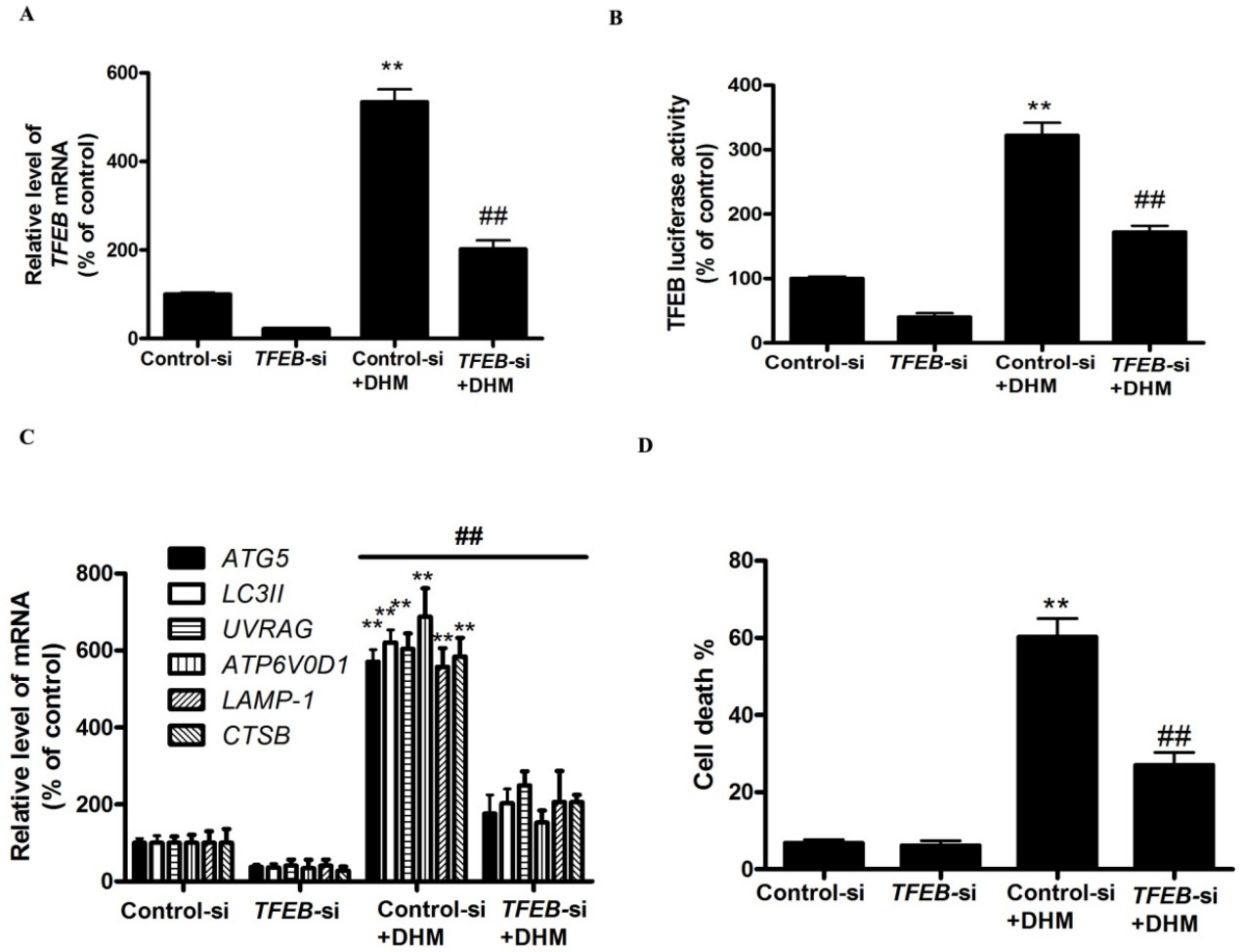
TFEB mediates DHM-induced autophagy. (A) The mRNA levels of *TFEB* were determined in *TFEB* siRNA-transfected A431 cells using RT-PCR. (B-C) A431 cells were treated with siRNA against *TFEB* or control siRNA and then incubated with 100 µM DHM for another 24 h before measuring the Luciferase activity and TFEB-target genes. (D) Trypan blue assay used to assess cell death. **P <0.01 versus the control group, ^##^P< 0.01 versus the Control-si+ DHM (100 µM) group (n=6).

To further confirm the molecular mechanism underlying modulation of autophagy by TFEB transcriptional factor in DHM-treated A431 cells, we examined the effect of TFEB gene silencing on DHM-induced autophagy in A431 cells. As shown in Fig. 3A and 3B, TFEB-siRNA abrogated DHM-induced the TFEB mRNA levels and luciferase activity. Notably, the inhibition of TFEB activity decreased DHM-induced autophagy-related genes expression (Fig. 3C). Additionally, the DHM-induced A431 cell death were also suppressed (Fig. 3D and S2C). These results suggested that DHM induced autophagic cell death by activating TFEB pathway in A431 cells.

### MALAT1 is an upstream signaling molecule that activates the TFEB-dependent autophagy pathway

**Figure 4:**
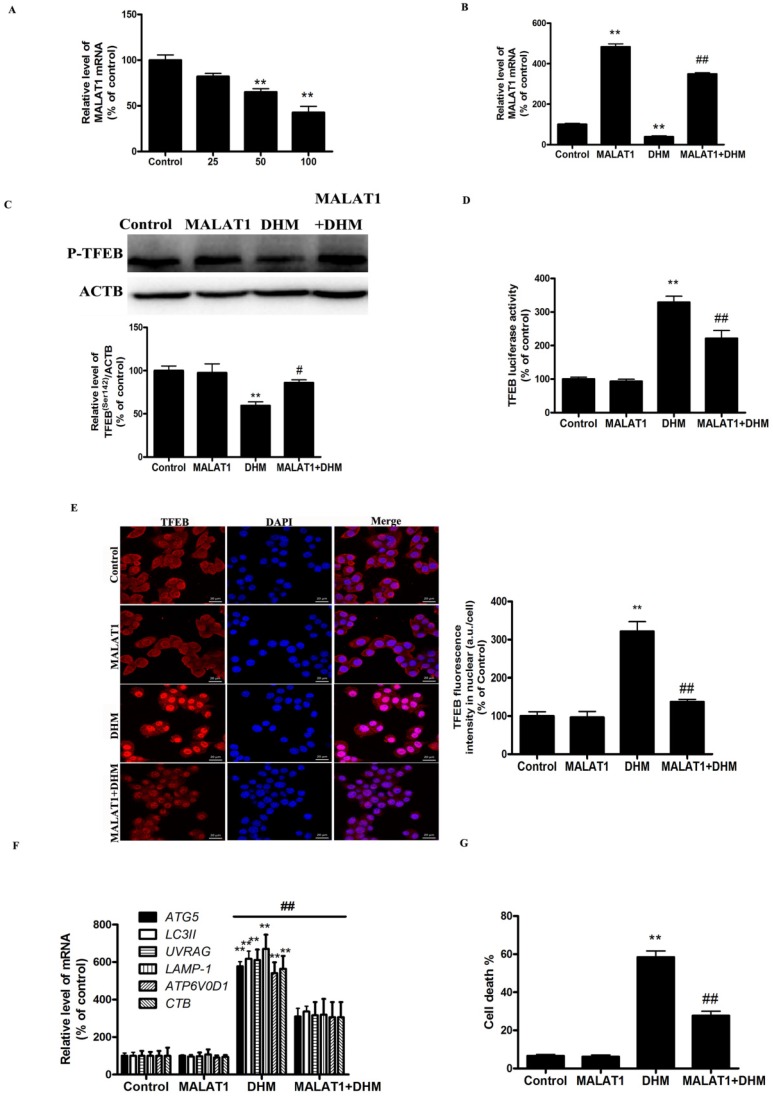
MALAT1 is an upstream signaling molecule that activates the TFEB-dependent autophagy pathway. (A) A431 cells were treated with different doses of DHM and then examined the *MALAT1* expression; The effect of *MALAT1* overexpression on TFEB-depended autophagy was studied. (B) The mRNA level of TFEB was detected by RT-PCR. (C) phosphorylation of TFEB protein was detected by western blotting; (D) The TFEB Luciferase activity was then measured. (E) The levels of nuclear TFEB were analyzed by Immunofluorescence; (F) The mRNA levels of TFEB-target genes were measured using RT-PCR. (G) Trypan blue assay used to assess cell death. * P<0.05, **P<0.01, versus the control group;^ #^ P<0.05, ^##^ P<0.01 versus the DHM (100 µM) group (n=6).

As shown in Fig. 4A, DHM induced a dose-dependent decrease in the level of MALAT1. We next examined the role of DHM-decreased MALAT1 in stimulating TFEB signaling. Here, MALAT1 overexpression significantly inhibited the activation of TFEB in DHM-treated cells, indicating that MALAT1 is an upstream signaling molecule that inactivates the TFEB pathway and cell death (Fig. 4B-G, and S2D). Taken together, these results showed that MALAT1 is involved in DHM-induced autophagy in A431 cells, and DHM-promoted TFEB signaling partly depends on MALAT1 inhibition.

### MALAT1 overexpression inhibited DHM-induced TFEB-depended autophagic cell death in vivo

**Figure 5:**
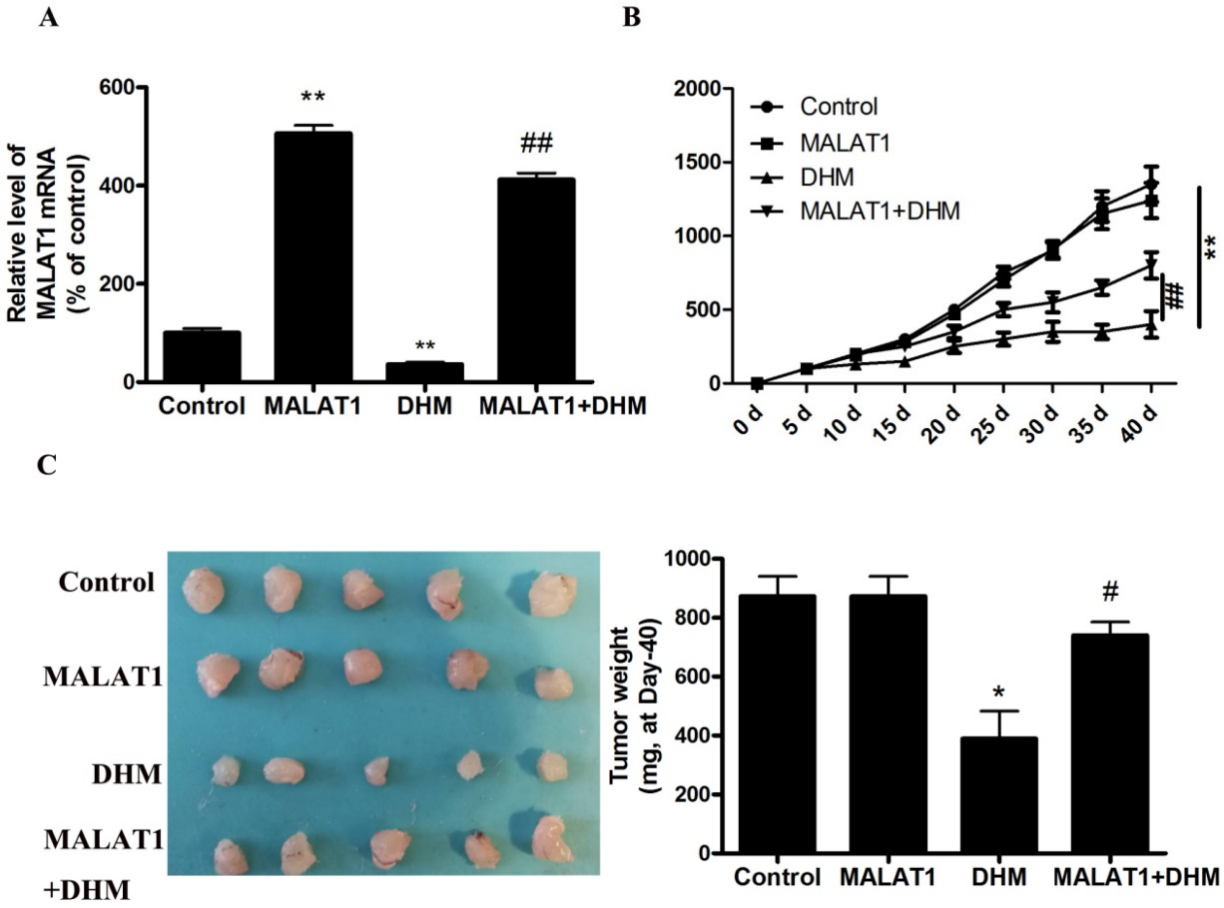
MALAT1 overexpression abolished anti-tumor effect of DHM *in vivo*. (A) The mRNA level of MALAT1 was detected by RT-PCR. (B-C) After excision from the mice, the xenografts were photographed, and the tumor volume and weight were measured. * P<0.05, **P<0.01, versus the control group;^ #^ P<0.05, ^##^ P<0.01 versus the DHM group (n=10).

**Figure 6:**
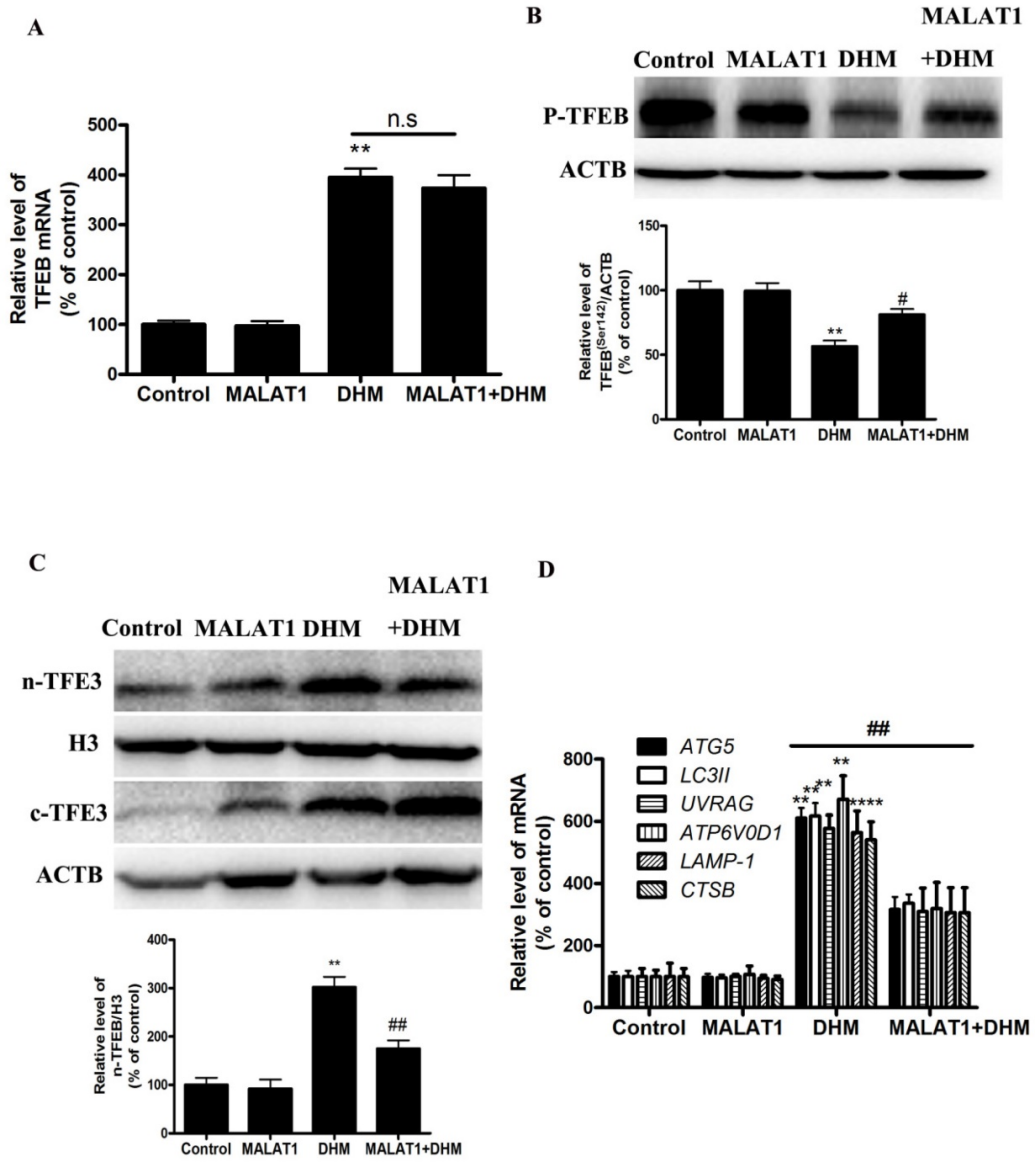
MALAT1 overexpression abolished DHM-induced TFEB-depended autophagic cell death *in vivo*. (A) The mRNA level of TFEB was detected by RT-PCR. (B) phosphorylation of TFEB (Ser142) protein was detected by western blotting; (C) The levels of cytoplasmic and nuclear TFEB protein were analyzed by Western blot; (D) The mRNA levels of TFEB-target genes were measured using RT-PCR. **P<0.01, versus the control group;^ #^ P<0.05, ^##^ P<0.01 versus the DHM group (n=10).

Based on the in vitro findings, we investigated whether the MALAT1 overexpression could suppress the antitumor effect of DHM in vivo. A xenograft tumor model was established by the subcutaneous inoculation of A431 cells into nude mice. As shown in Figure 5, the antitumor ability of DHM combined with MALAT1 overexpression was lower than that of DHM alone; Furthermore, MALAT1 overexpression increased TFEB (Ser142) phosphorylation, prevented TFEB transcription activity and nuclear translocation but not TFEB expression, suppressed autophagy-related genes expression (Fig. 6). Taken together, MALAT1 overexpression also abolished DHM-induced TFEB-depended autophagic cell death *in vivo*.

## Discussion

DHM is known to inhibit the growth of cancer cells and to exhibit certain biological effects, but the mechanisms of these actions are still only partially understood. The present study was undertaken to shed more light on the mechanisms by which DHM exerts its anti-proliferative properties in cultured CSCC cells. For the first time, we have demonstrated that (**i**) DHM induces autophagic cell death in A431 cells; (**ii**) DHM inhibited TFEB (Ser142) phosphorylation subsequently to activate TFEB nuclear translocation and increase TFEB reporter activity, which contributed to the expression of autophagy-related genes; and (**iii**) DHM increased TFEB-dependent autophagic cell death by disturbing *MALAT1* homeostasis.

Studies on cancer treatment have identified autophagy activation as a consequence of chemotherapy or radiotherapy; however, the role that autophagy plays in cancer progression is varied. Generally, autophagy is considered as a prosurvival mechanism in cancer cells by removing damaged organelles and recycling nutrients upon anticancer treatment 17. For example, DHM induces cytoprotective autophagy in human melanoma cells and head and neck squamous cell carcinoma cells 15,18. However, a recent remarkable finding is that autophagy induced by certain chemotherapeutic agents may have a suppressive role in cancer cells, revealing two additional functional forms of autophagy, of which one is the cytotoxic function that results in autophagic cell death 19, the other is the cytostatic function that may inhibit cell proliferation in an apoptosis-independent way^20^. Our results revealed that, autophagy inhibitor 3-MA or ATG5*-*siRNA could avert the effect of DHM on cell death, which indicated that DHM-induced autophagic cell death in CSCC cells.

We also examined the potential mechanisms underlying DHM-induced autophagy. The MiTF/TFE family (MITF, TFEB, TFE3, and TFEC) of transcription factors is emerging as global regulators of cancer cell survival and energy metabolism through the promotion of autophagy 21. MITF, TFEB, and TFE3 show a more ubiquitous pattern of expression and were detected in multiple cell types, whereas TFEC expression is restricted to cells of myeloid origin 22. This is the reason we did not measure TFEC expression in our model. Interestingly, in our study, DHM treatment increased TFEB but not TFE3 or MITF expressions, which further confirmed the important role of TFEB in DHM-mediated therapy of CSCC (Figure S3). The transcription factor TFEB acts as a master regulator of cellular clearance through enhancement of the autophagy-lysosome pathway, including lysosomal biogenesis, expression of autophagy genes and lysosomal proteostasis 23. The function of TFEB following cell death is still controversial, with both beneficial and detrimental roles suggested. Recently, Wen-Xing Ding group identified that activating TFEB protected against EtOH-induced steatosis and liver injury 24. However, Wei Li et al. have shown that clomiphene citrate induces nuclear translocation of the TFEB transcription factor and triggers apoptosis by enhancing lysosomal membrane permeabilization 25. It has also been shown that TFEB is activated, translocated to the nucleus and mediates osteosarcoma cells death via increasing autophagy-related gene in response to arsenic trioxide toxicity 19. Consistent with these findings, our study has revealed that TFEB-siRNA blocks DHM-induced CSCC cell death, via decreasing autophagy-related genes.

Metastasis-associated lung adenocarcinoma transcript 1 (MALAT1) was first identified as a member of the lncRNAs that is highly expressed in several types of cancer 26. The expression level of MALAT1 is closely related to melanoma progression, as, for instance, silence of MALAT1 would delay tumour growth of melanoma, indicating the potential of MALAT1 as a theranostic target of melanoma treatment 27,28. Moreover, MALAT1 promotes the proliferation and metastasis of gallbladder cancer cells by activating the ERK/MAPK pathway 29. In our study, DHM decreased *MALAT1* expression. Moreover, An RNA immunoprecipitation (RIP) assay was conducted using an anti‐TFEB antibody with high specificity for TFEB to verify the interaction between MALAT1 and TFEB. The results showed an obvious enrichment of MALAT1 when using the TFEB antibody compared with that using an IgG antibody in A431 cells, and which indicated binding between MALAT1 and TFEB (Figure S4). Thus, it may be predicted that *MALAT1* might be required for TFEB nuclear translocation in DHM-treated A431 cells. Herein, we next examined a possible correlation between DHM-inhibited *MALAT1* expression and TFEB activation. Our findings show that *MALAT1* overexpression abrogated the effects of DHM on the de-phosphorylation of TFEB. Based on these results, we suggested that lncRNA* MALAT1* is an important cellular mediator that triggers the TFEB-dependent pathway after the administration of DHM, which promotes cell death in human CSCC, and MALAT1 may regulate TFEB gene expression at both the transcriptional and posttranscriptional level.

In summary, DHM induces TFEB-dependent autophagic cell death by disturbing *MALAT1* homeostasis in A431 Cells. Thus, these compelling evidences indicated that DHM may be a potential and effective candidate against human CSCC for its well anticancer efficiency and high safety.

## Supplementary Material

Supplementary figures and tables.
