# Supplementary material for: Dihydromyricetin induced lncRNA MALAT1-TFEB-dependent autophagic cell death in cutaneous squamous cell carcinoma
Source: J Cancer. 2019 Jul 10;10(18):4245–55. doi: 10.7150/jca.32807 (PMC6691703; doi:10.7150/jca.32807)
Supplement: Supplementary file 1 — Supplementary figures and tables. [file jcav10p4245s1.pdf]

Figure S1

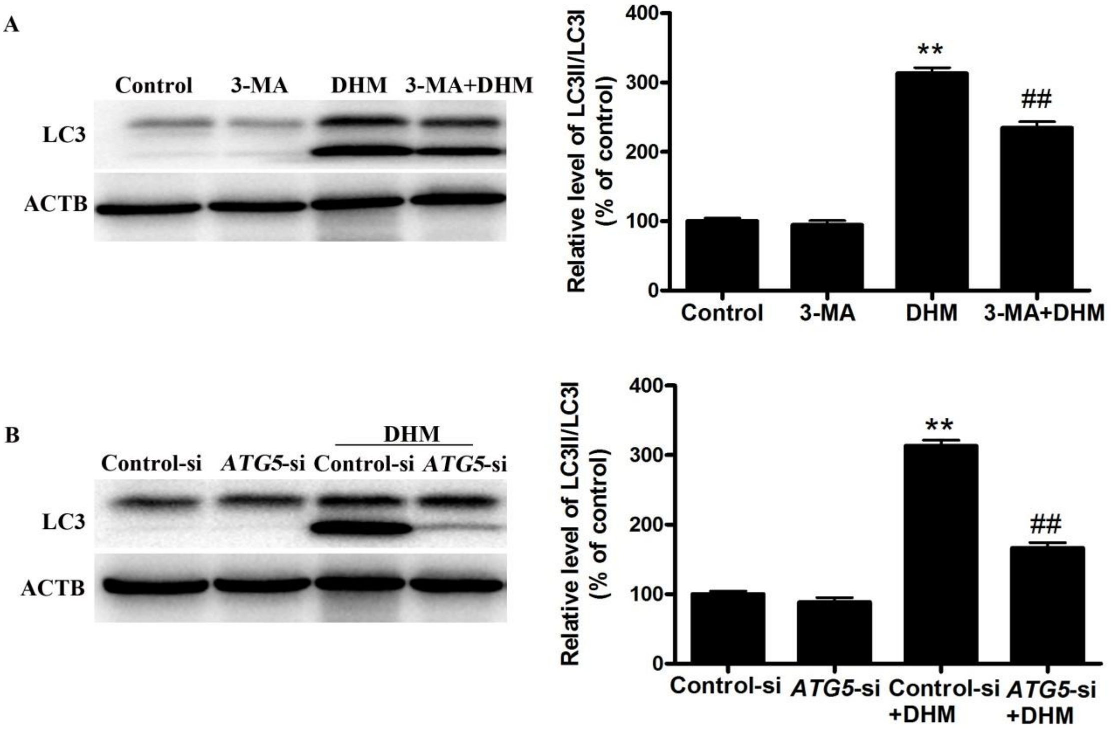

Figure S1. 3-MA or si-ATG5 could efficiently inhibit autophagy. A431 cells were preincubated with (A-B) 3-MA (2 mM) for 2 h or *ATG5*-si for 24 h and then treated with 100 μM DHM for another 24 h, then the LC3II levels were then assessed by western blotting.

Figure S2

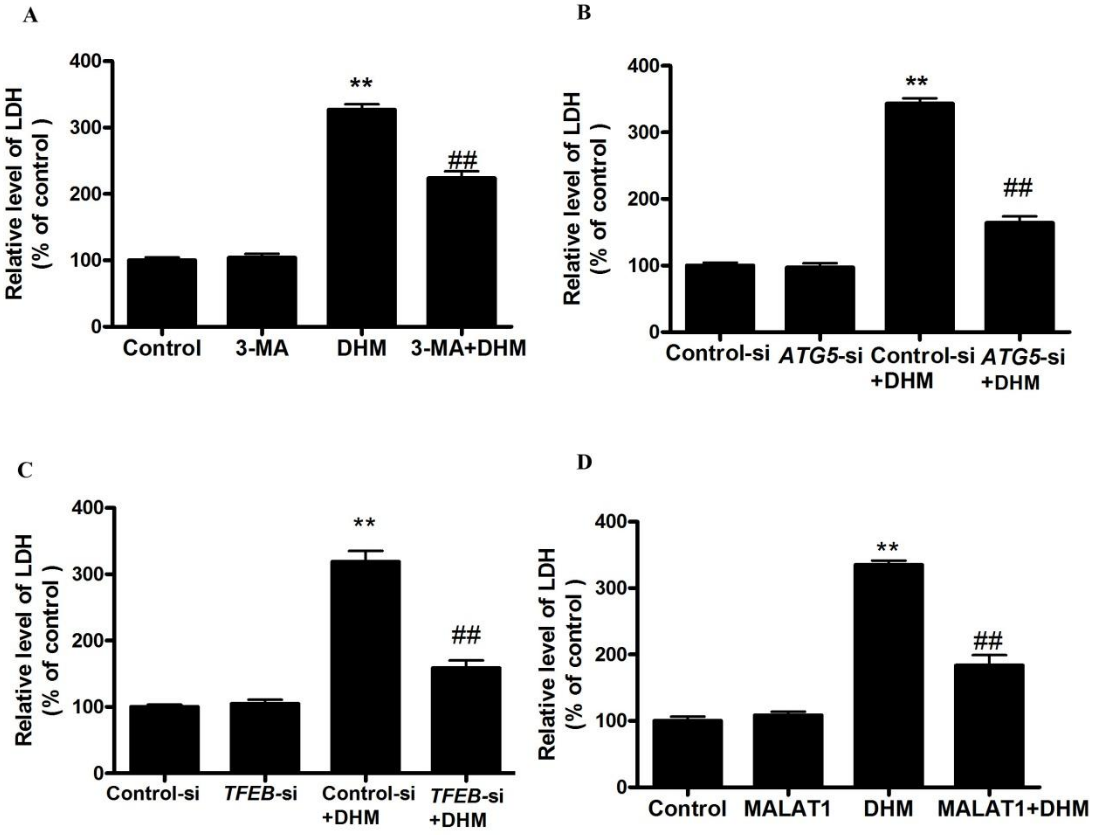

Figure S2. An LDH assay was used to assess cell death. A431 cells were preincubated with (A) 3-MA (2 mM) for 2 h, or (B) *ATG5*-si, or (C) *TFEB*-si, or (D) MALAT1 overexpression for 24 h and then treated with 100 μM DHM for another 24 h, then an LDH assay was used to assess cell death.

Figure S3

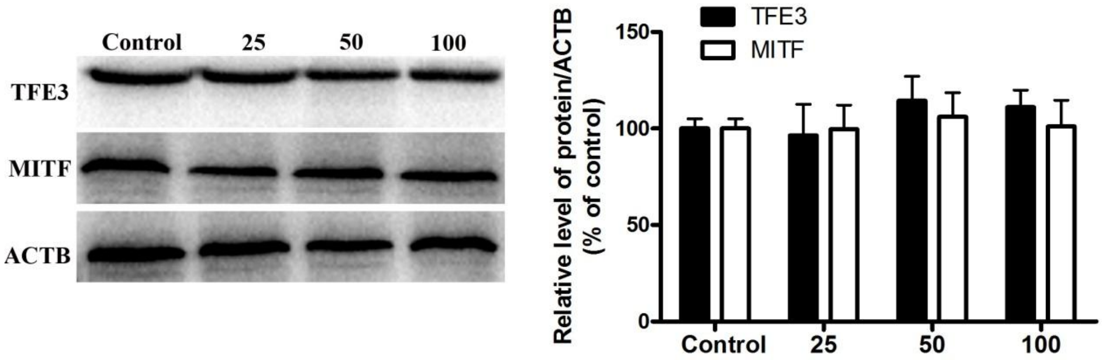

Figure S3. DHM treatment did not change the TFE3 or MITF expressions. A431 cells were treated with different concentrations of DHM for 24 h, and the expression levels of the TFE3 and MITF were then assessed by western blotting.

Figure S4

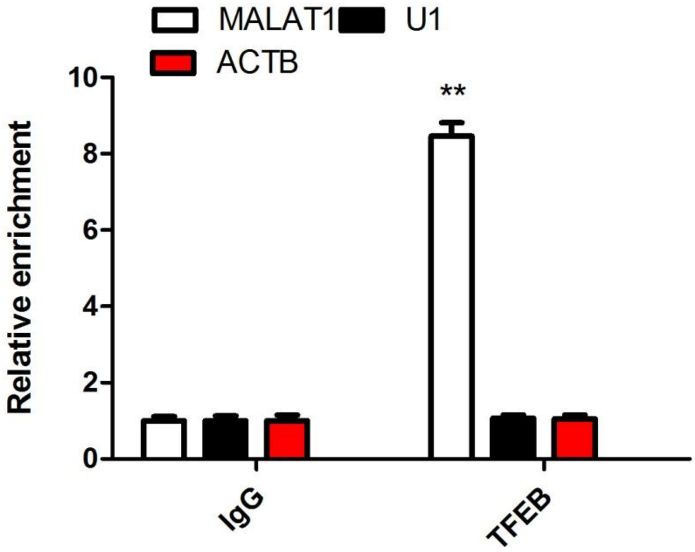

Figure S4. MALAT1 interacted with TFEB in A431 **cells.** RIP assay showed that MALAT1 was preferably enriched with TFEB antibody compared to that in IgG group in A431 cells. **P <0.01versus the control group (n=6).

Table S1: Antibodies used for the western blot

Antigen	Dilution	Catalogue number	Supplier
LC3	1:1000	L7543	Sigma
P62/SQSTM1	1:1000	ab91526	Abcam
TFEB	1:1000	PA1-31552	Thermo fisher
MITF	1:1000	ab20663	Abcam
TFE3	1:1000	ab173928	Abcam
p-TFEB$^{(Ser142)}$	1:500	ABE1971	Merck
ACTB	1:5000	A1978	Sigma
H3	1:1000	SAB4500352	Sigma
anti-mouse (secondary antibody)	1:1000	A0208	Beyotime Company
anti-rabbit (secondary antibody)	1:1000	A0216	Beyotime Company

Table S2: Sequences of primers used in quantitative RT-PCR

Target gene	Primer	Nucleotide sequence
TFEB	F	5’-ACCTGTCCGAGACCTATGGG-3’
	R	5’-CGTCCAGACGCATAATGTTGTC-3’
MAP1LC3B	F	5’-AGCAGCATCCAACCAAAATC-3’
	R	5’-CTGTGTCCGTTCACCAACAG-3’
ATP6V0D1	F	5’-TTCCCGGAGCTTTACTTTAACG-3’
	R	5’-CAAGTCCTCTAGCGTCTCGC-3’
UVRAG	F	5’-GGCGTCTTCGACATCTTCGG-3’
	R	5’-GACGGTCTGGCATAATTCCAAA-3’
CTSB	F	5’-GAGCTGGTCAACTATGTCAACA-3’
	R	5’-GCTCATGTCCACGTTGTAGAAGT-3’
LAMP-1	F	5’-TCTCAGTGAACTACGACACCA-3’
	R	5’-AGTGTATGTCCTCTTCCAAAAGC-3’
ATG5	F	5’-AAAGATGTGCTTCGAGATGTGT-3’
	R	5’-CACTTTGTCAGTTACCAACGTCA-3’
ACTB	F	5’-CATGTACGTTGCTATCCAGGC-3’
	R	5’-CTCCTTAATGTCACGCACGAT-3’
